# Nanotoxicology: Gangwal et al. Respond

**DOI:** 10.1289/ehp.1104320R

**Published:** 2012-01-01

**Authors:** Sumit Gangwal, Elaine A. Cohen Hubal

**Affiliations:** National Center for Computational Toxicology Office of Research and Development, U.S. Environmental Protection Agency, Research Triangle Park, North Carolina, E-mail: gangwal.sumit@epa.gov

We appreciate the letter from Oberdörster commenting on the importance of careful selection of *in vitro* doses for nanomaterial (NM) toxicity testing and his assessment of our article (Gangwal et al. 2011). Because the objective of our study was to use limited data on potential human occupational exposure to NMs to identify bounding limits for toxicity testing, we believe our conclusions and Oberdörster’s views to be generally aligned.

Our article described how to apply sparse NM exposure information from manufacturing and R&D (research and development) settings and relevant particle dosimetry model inputs, based on a report of the International Commission on Radiological Protection (1994), to estimate NM mass retained in the alveolar region of the human lung. Modeled alveolar lung surface concentrations (micrograms per square centimeter) were then used to estimate bounding *in vitro* NM solution concentrations (micrograms per milliliter) representative of short-term (24-hr) and long-term (full occupational lifetime of 45 years) exposure scenarios. In comparing our rough “equivalent” estimates obtained based on lifetime exposure to concentrations currently being used for *in vitro* testing, we indeed intended to highlight that such concentrations represent a high end bounding limit, as Oberdörster has emphasized. Equivalent *in vitro* concentrations based on a 24-hr scenario are intended to represent more realistic short-term exposures.

We agree with Oberdörster that our article (Gangwal et al. 2011) should not be viewed as justification for using very high NM *in vitro* testing concentrations. Rather, we demonstrate the importance of understanding *in vitro* concentrations in the context of the potential for human NM exposure to improve study design and facilitate interpretation of testing results. For NMs currently being tested in the U.S. Environmental Protection Agency’s (EPA) ToxCast project (Dix et al. 2007), we are in fact evaluating multiple concentrations based on consideration of potential exposure and generally have set NM testing concentrations to range from below the 24-hr inhalation exposure equivalent to the full working lifetime equivalent.

As we note in our article (Gangwal et al. 2011) and as Oberdörster has further emphasized, there are significant uncertainties associated with our estimates of exposure and associated dosing concentrations. These include uncertainties associated with screening-level tools available for modeling deposition of engineered nanomaterials and with our understanding of characteristics and properties of materials found in the human environment. In the interest of mining available tools to inform design of toxicity tests for immediate use, we did opt to make significant simplifying assumptions related to particle characteristics and to apply a version of the MPPD model adapted by the developers for application to nanofibers/nanotubes (National Institute for Occupational Safety and Health 2008). The modeled alveolar mass retained for CNTs based on more realistic, short 24-hr inhalation exposure duration is available online (U.S. EPA 2011).

One point that Oberdörster missed in our article (Gangwal et al. 2011) is that we calculated alveolar lung surface concentration using the same low estimate of human alveolar surface area for both the full working lifetime and the 24-hr exposure duration, and thus calculations for both exposure scenarios may be lower by approximately one order of magnitude.

We are pleased that our framework for using available exposure information to inform selection of *in vitro* toxicity testing concentrations is generating important discussion. We believe the issues and limitations raised in our article and by Oberdörster are important and demonstrate a critical need for continuing research to understand the potential for human exposure to engineered nanomaterials and to design environmentally relevant toxicity testing schemes.
